# DNA Hypermethylation Downregulates Telomerase Reverse Transcriptase (TERT) during *H. pylori*-Induced Chronic Inflammation

**DOI:** 10.1155/2019/5415761

**Published:** 2019-12-31

**Authors:** Françoise I. Bussière, Valérie Michel, Julien Fernandes, Lionel Costa, Vania Camilo, Giulia Nigro, Hilde De Reuse, Laurence Fiette, Eliette Touati

**Affiliations:** ^1^Institut Pasteur, Unit of Helicobacter Pathogenesis, CNRS ERL6002, 25-28 Rue du Dr Roux, 75724 Paris cedex 15, France; ^2^Institut Pasteur, Unit of Molecular Microbial Pathogenesis, INSERM U1202, 25-28 Rue du Dr Roux, 75724 Paris cedex 15, France; ^3^Institut Pasteur, Unit of Human Histopathology and Animal Model, 25-28 Rue du Dr Roux, 75724 Paris cedex 15, France

## Abstract

*Helicobacter pylori* infection causes chronic gastritis and is the major risk factor of gastric cancer. *H. pylori* induces a chronic inflammation-producing reactive oxygen species (ROS) which is a source of chromosome instabilities and contributes to the development of malignancy. *H. pylori* also promotes DNA hypermethylation, known to dysregulate essential genes that maintain genetic stability. The maintenance of telomere length by telomerase is essential for chromosome integrity. Telomerase reverse transcriptase (TERT) is the catalytic component of telomerase activity and an important target during host-pathogen interaction. We aimed to investigate the consequences of *H. pylori* on the regulation of *TERT* gene expression and telomerase activity. *In vitro*, *hTERT* mRNA levels and telomerase activity were analysed in *H. pylori*-infected human gastric epithelial cells. In addition, C57BL/6 and INS-GAS mice were used to investigate the influence of *H. pylori*-induced inflammation on TERT levels. Our data demonstrated that, *in vitro*, *H. pylori* inhibits *TERT* gene expression and decreases the telomerase activity. The exposure of cells to lycopene, an antioxidant compound, restores TERT levels in infected cells, indicating that ROS are implicated in this downregulation. *In vivo*, fewer TERT-positive cells are observed in gastric tissues of infected mice compared to uninfected, more predominantly in the vicinity of large aggregates of lymphocytes, suggesting an inflammation-mediated regulation. Furthermore, *H. pylori* appears to downregulate *TERT* gene expression through DNA hypermethylation as shown by the restoration of *TERT* transcript levels in cells treated with 5′-azacytidine, an inhibitor of DNA methylation. This was confirmed in infected mice, by PCR-methylation assay of the *TERT* gene promoter. Our data unraveled a novel way for *H. pylori* to promote genome instabilities through the inhibition of TERT levels and telomerase activity. This mechanism could play an important role in the early steps of gastric carcinogenesis.

## 1. Introduction

*Helicobacter pylori* is a gastric pathogen that infects half of the human population worldwide. This bacterium is responsible for chronic inflammation and gastroduodenal diseases, including gastric adenocarcinoma and mucosa-associated lymphoid tissue (MALT) lymphoma [1, 2]. *H. pylori* is, to date, the first and only bacterium identified as a type I carcinogenic agent in humans [3]. The complex interplay between bacterial, host, and environmental factors plays a fundamental role in the development of gastric cancer lesions. Prolonged inflammation and long-term persistence of *H. pylori* contribute to gastric carcinogenesis, *via* dysregulation of signaling pathways, cell proliferation, and chromosome instability [4, 5]. *H. pylori* is an efficient inducer of DNA damage such as DNA double-strand breaks (DSBs) and mutations in the nuclear and mitochondrial DNA [6–9]. The genotoxic activity of *H. pylori* infection is largely associated with chronic inflammation of the gastric mucosa and the resulting oxidative stress, leading to a harmful environment for the host and promotion of carcinogenesis [10]. Oxidative stress is a source of DNA damage and telomere shortening [11]. Recently, a unique *H. pylori*-induced pattern of DNA damage accumulation has been shown preferentially in transcribed regions and in proximal regions of telomeres [12]. *H. pylori* is also a source of aberrant DNA methylation in the host cells [5, 13]. In a previous study, we reported that *H. pylori* inhibits the expression of the transcription factors *USF1* and *USF2* (upstream stimulating factors 1 and 2) genes, by DNA hypermethylation of their promoter region [14]. USF1 and USF2 regulate among others the transcription of *TERT* coding for the telomerase reverse transcriptase (TERT), the major component of telomerase [15, 16].

Telomerase maintains the telomere length essential for chromosome stability and integrity [17]. This ribonucleoprotein is also involved in cell transformation and lymphocyte activation [18]. The telomerase complex includes the reverse transcriptase catalytic subunit (TERT) and a telomerase RNA component (TERC). It elongates telomere ends by adding TTAGGG repeats and prevents telomere shortening during cell division. It is regulated mainly at the TERT transcriptional level [15]. In addition to telomere elongation, hTERT (human TERT) plays a role in diverse cellular processes, such as the transcriptional modulation of Wnt-*β*-catenin signaling pathway and DNA damage response [19]. Importantly, hTERT is a strategic target for bacterial infection, as previously reported for *Listeria monocytogenes* which promotes hTERT degradation [20].

Telomerase activation is an essential event during the carcinogenesis process, allowing cells to proliferate indefinitely and to avoid apoptosis. In most advanced carcinomas and soft cancer tissues, telomerase expression is upregulated [21]. Increased *hTERT* transcription is observed in more than 85% of tumor cells and is lower in most somatic cells [22]. Slightly elevated levels of *TERT* mRNA and protein were also reported in 45 to 50% of intestinal metaplasia and gastric ulcer cases, and 79% of gastric cancer showed higher TERT levels [23]. In *H. pylori*-positive patients, telomere reduction has been reported in the gastric mucosa [24]. Moreover, reactive oxygen species (ROS) overproduction during *H. pylori*-induced chronic inflammation has been demonstrated as a cause for telomere shortening [25].

In the present study, the consequences of *H. pylori* infection on TERT were investigated *in vitro* in human gastric epithelial cells and in mouse models at an early step of the development of gastric preneoplasia. Our data reveal that *H. pylori* infection downregulates *TERT* gene expression through DNA methylation and thus impairs telomerase activity. Given the role of telomerase in the control of chromosome integrity and epithelial cancer development, these mechanisms could promote the transition between the chronic stage of the infection and the development of neoplasia.

## 2. Materials and Methods

### 2.1. Bacterial Strains and Growth Conditions

*H. pylori* strains B38, isolated from a MALT lymphoma patient [26, 27] (obtained from Pr F. Mégraud, Bordeaux, France), 7.13 and its derivative mutants *∆cagA* and *∆cagE* [28] (obtained from Pr RM Peek Jr, Vanderbilt, USA), as well as SS1 [29], a mouse-adapted human strain, were grown on 10% blood agar under microaerobic conditions with the following antibiotics-antifungal cocktail: amphotericin B 2.5 *μ*g·ml^−1^, polymyxin B 0.31 *μ*g·ml^−1^, trimethoprim 6.25 *μ*g·ml^−1^, and vancomycin 12.5 *μ*g·ml^−1^. Bacteria lysates were obtained by passage of bacterial suspensions through a French pressure cell as previously described [30]. Protein concentration of supernatants was measured with the DC Protein assay (Biorad, Hercules, CA).

### 2.2. Cell Culture and Infection

Human adenocarcinoma gastric cell lines AGS (CRl-1739 and ATCC-LGC), MKN45 (CVCL_0434), and KatoIII (CVCL_0371), a gift from Dr C. Figueiredo, Porto, Portugal, were grown in DMEM medium with 10% fetal bovine serum and 1% penicillin-streptomycin (Life Technologies Corporation, Carlsbad, CA) for AGS and MKN45 cells and with 20% fetal bovine serum for KatoIII cells (Life Technologies Corporation, Carlsbad, CA, USA). Bacteria were added at a multiplicity of infection (MOI) of 20, 50, and 100 bacteria per cell for 12, 24, and 48 h. To inhibit DNA methylation, cells were treated with 5′-azacytidine 1 *μ*M (Sigma Chemical Co., St. Louis, MO) for 3 days, prior to infection for 48 h. To avoid any effect of 5′-azacytidine on bacteria and as similar results were obtained with live bacteria and lysate on *TERT* expression, cells were then treated with *H. pylori* B38 lysate (20 *μ*g·ml^−1^), equivalent to 10^8^ bacteria for 10^6^ epithelial cells. To inhibit ROS production, cells were treated with lycopene 5 *μ*M (Sigma Chemical Co., St. Louis, MO), dissolved in dimethyl sulfoxide (DMSO) 25%, prior to *H. pylori* infection, as previously described [31]. Control cells were incubated with DMSO 2.5% corresponding to the final concentration of DMSO in the vehicle solvent in the culture medium.

For gene expression analysis, total RNA was extracted from cells, as previously described [14]. Proteins were isolated by lysis of cells in NP40 buffer and analysed by Western blot using antibodies against TERT (sc-7212; 1/200; Santa Cruz Biotechnology, CA, USA) and GAPDH (sc-25778; 1/200; Santa Cruz Biotechnology, CA, USA).

### 2.3. Measurement of Intracellular ROS

The production of ROS was assessed using the ROS-sensitive fluorescent probe 2′,7′-dichlorodihydrofluorescein diacetate (H_2_-DCF-DA) (Sigma Aldrich) as previously described [32]. In brief, the H_2_-DCF-DA probe freely enters the cells where it is cleaved to nonfluorescent and impermeant product, which is later oxidized by ROS to DCF, a fluorescent compound. For these assays, 4 × 10^4^ MKN45 cells were plated in 96-well plates, in quintuplicate. The following day, these cells were treated with 10 *μ*M H_2_-DCF-DA for 30 minutes at 37°C and washed 3 times with PBS. Afterwards, cells were exposed for 24 h to either different concentrations of bacterial extracts (20 *μ*g·ml^−1^, 50 *μ*g·ml^−1^, or 100 *μ*g·ml^−1^) obtained from the 7.13 *H. pylori* strain or the vehicle control. H_2_O_2_ (5 mM) was used as a positive control, and WT MKN45 cells were used as a reference for ROS production at basal levels. DCF fluorescence was measured using an excitation/emission wavelength of 488/530 nm with an Infinite M200PRO microplate reader (Tecan).

### 2.4. Animal Infection

#### 2.4.1. Ethical Statement

Mouse experiments were carried out in strict accordance with the recommendations in the Specific Guide for the Care and Use of Laboratory Animals of the Institut Pasteur, according to the European Directives (2010/63/UE). The project was approved by the Comité d'Éthique en Expérimentation Animale (CETEA), Institut Pasteur and the Ministère de l'Enseignement Supérieur et de la Recherche, France (Ref 00317.02).

Two different mouse models were used in this study. The first model consists of six-week-old specific pathogen-free (SPF) C57BL/6 male mice (Charles Rivers, France), which were orogastrically infected with *H. pylori* SS1 (10^7^ cfu/100 *μ*l) for 12 and 18 months (*n* = 6/group). Control mice received peptone trypsin broth alone. The second model corresponds to INS-GAS mice, which are transgenic for the human gastrin, leading to an exacerbated development of gastric neoplasia in the presence of *H. pylori,* as early as 7–9 months after infection [33]. Three couples of SPF INS-GAS/FVB mice were kindly provided by Pr. T.C. Wang (Columbia University, NY, USA) and bred at the animal facility of the Institut Pasteur. Six-week-old INS-GAS/FVB male mice (*n* = 6/group) were infected with *H. pylori* SS1 as described above, for 8 months. At each time point, mice were sacrificed and stomachs were collected and used for the quantification of gastric colonization, histological analysis, RNA extraction, and genomic DNA isolation as previously described [6, 14].

### 2.5. PCR and Real-Time qPCR Analysis

RNA extraction and cDNA synthesis were performed as previously described [14]. Gene expression in human gastric epithelial cells was measured by real-time quantitative PCR (qPCR) analysis using TaqMan® Gene Expression Assays (Applied Biosystems, Thermo Fischer Scientific, France). TaqMan gene expression primers were *hTERT* (Hs99999022_m1) and *18S* (Hs99999901_s1) (Applied Biosystems, Thermo Fischer Scientific, France) as the endogenous control. For mouse analysis, primers were *mTERT* (Mn01352136-m1) and *GAPDH* (Mn99999915-g1) as an endogenous control (Applied Biosystems, Thermo Fischer Scientific, France). Quantitative PCR was performed in triplicate. The expression of hTERT and mTERT was normalized to Ct values obtained for *18S* and GAPDH, respectively, using the ΔCt formula: Ct gene–Ct housekeeping gene. For each experiment, fold changes for *TERT* RNA levels were determined from this calculation for infected samples to the uninfected control 2^−(ΔΔCt)^, for at least two independent biological and three technical replicates.

### 2.6. Detection of Protein Levels by Western Blot

After coculture with *H. pylori*, cells were lysed in NP-40 buffer containing protease inhibitors; 20 *μ*g per lane were separated on a 12% Mini-PROTEAN® TGX Stain-Free™ Precast Gel (BioRad) and transferred onto Trans-Blot® Turbo™ Midi PVDF Transfer Packs using a Trans-Blot® Turbo™ Transfer System (BioRad). TERT (H-231) antibodies (Ref sc-7212, Santa Cruz Biotechnology, CA, USA; dilution 1/500) and GAPDH ((FL-335) sc-25778, Santa Cruz Biotechnology, CA, USA; 1/100) were used, followed by a goat anti-rabbit IgG-HRP (sc-2054, Santa Cruz Biotechnology, CA, USA; 1/10000). Detection was performed using the Clarity™ Western ECL Substrate (BioRad) and revealed using a ChemiDoc XRS (Bio-Rad). Western blot data were quantified by densitometry using Image Lab software (Bio-Rad).

### 2.7. Telomerase Repeat Amplification Protocol Assay (TRAP Assay)

Telomerase activity was analysed by TRAP assay [34] using a TRAPeze® telomerase detection kit (Chemicon-Millipore, Billerica, MA), according to the manufacturer's instructions. In brief, the telomerase activity in cell extracts (150 ng) was evaluated by its ability to extend the 3′ end of an oligonucleotide substrate with telomeric repeats (GGTTAG). The primary telomerase products were then amplified by PCR, generating a ladder of products with 6 base increments starting at 50 nucleotides length. Reaction products were detected by electrophoresis on 12.5% nondenaturing polyacrylamide gel (PAGE) stained with SYBR® Green followed by UV detection (Gel Doc System, Bio-Rad).

### 2.8. Histology and Immunohistochemistry

For both mouse models, C57BL/6 and INS-GAS, stomachs from uninfected or *H. pylori* SS1-infected mice were fixed in 4% formalin and then embedded in low-melting point paraffin (Poly Ethylene Glycol Distearate; Sigma, USA). Four *μ*m thick paraffin sections were deparaffinised in absolute ethanol, air-dried, and then stained with hematoxylin-eosin (H&E) or used for immunolabeling. Immunostaining of B and T lymphocytes was performed using anti-CD45R (RM2600, 1/40, Invitrogen, Carlsbad, CA, USA) and anti-CD3 (A0452; 1/75, DAKO, Carpinteria, CA, USA), respectively. *In situ* expression of TERT was visualized by immunodetection with a rabbit polyclonal antibody against telomerase catalytic subunit (Ref 600-401-252; 1 : 200; Rockland Immunochemicals Inc., Gilbertsville, PA, USA). The staining was revealed using peroxidase detection as previously described [6, 14].

### 2.9. Determination of DNA Methylation Status in the Mouse TERT-Promoter Region

**Figure 1:**
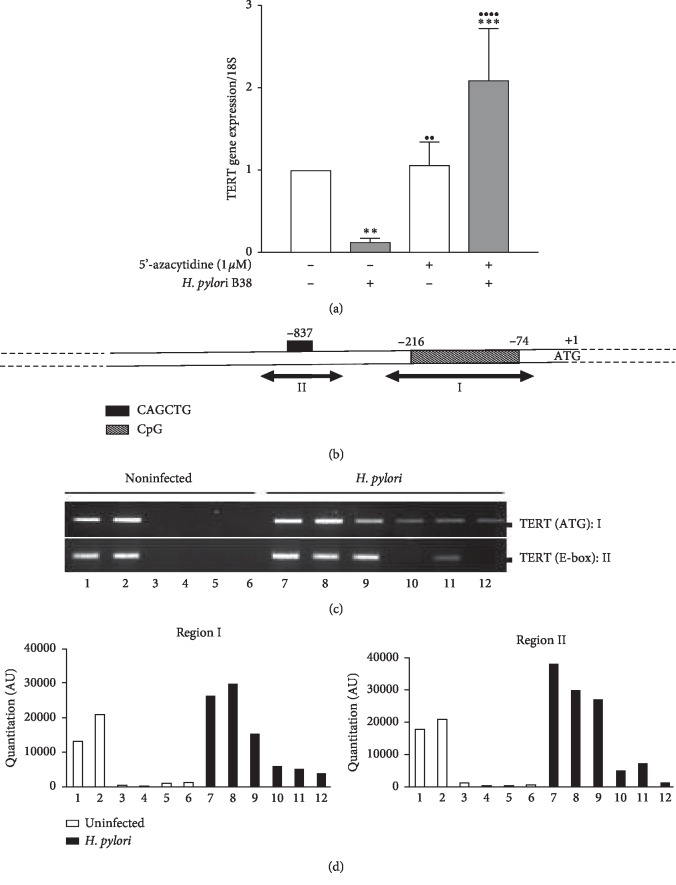
DNA methylation downregulates *hTERT* gene expression during *H. pylori* infection. (a) AGS gastric epithelial cells were treated with 5′-azacytidine (1 *μ*M) for 3 days before incubation with *H. pylori* B38 lysate (20 *μ*g·ml^−1^) for 48 h. Quantification of *hTERT* gene expression was performed by real-time qPCR. 5′-azacytidine treatment restores *hTERT* gene expression in cells stimulated with *H. pylori* B38 lysate. Results are expressed as mean ± SD of at least 2 independent experiments in duplicate. *p* < 0.0001; one-way ANOVA Kruskal–Wallis test followed by Dunn's multiple comparison (infected *versus* uninfected ^*∗∗*^*p* < 0.01; ^*∗∗∗*^*p* < 0.001; infected *versus* azacytidine treated ± infection ^*••*^*p* < 0.01; ^*••••*^*p* < 0.0001) (b) Structure of the *mTERT* gene-promoter region in mice, containing a CpG island (hatched box, I and E-box element (black box, II)). (c) DNA methylation status of *mTERT*-promoter regions analysed by promoter methylation PCR assay, on genomic DNA isolated from the gastric mucosa of *H. pylori* SS1-infected (18 months) and uninfected mice, as described in the *Experimental procedures*. A representative gel of amplified methylated DNA is reported (upper panel) with each well corresponding to one mouse. (d) Quantification for each amplified methylated DNA fragment using BIO-PROFIL Bio-1D++ (Biosystems) software (lower panel), showing *H. pylori*-induced DNA hypermethylation in both *mTERT*-promoter regions I and II.

Two distinct regions of the *mTERT*-promoter region were selected between nucleotides −7 to −326 (segment I) and −791 to −1028 (segment II) (see Figure 1(b)). Segment I corresponds to a CpG island region including GC boxes and a noncanonical E-box (−197 to −202) [35]. Segment II presents a canonical E-box sequence at position −837. DNA methylation status was analysed using the Promoter Methylation PCR assay (Panomics, Redwood City, CA). Genomic DNA was extracted from 18-month-infected and uninfected mouse stomachs as previously described [6] and digested with *Ban*II restriction enzyme (New England Biolabs, Ipswich, MA). The methylated DNA was isolated according to the manufacturer's instructions; segments I and II were amplified by PCR using the following primers: 5′-GCCCGAGAAGCATTCTGTAG-3′ and 5′-CACTGAGAGTCCACGACGAA-3′ for the segment I, and 5′-GAAAGCTGAAGGCACCAAAG-3′ and 5′-GATGGCAGCTCTGCTAGGTT-3′ for the segment II (GenBank NG_055506.1). The PCR products were detected by agarose gel electrophoresis (Gel Doc System, Bio-Rad), and the band intensities were quantified by using Quantity One software (Bio-Rad).

### 2.10. Statistical Analysis

Statistical analysis was performed using the Student's *t* test or Mann–Whitney test, after being assessed for normality of samples distribution, for comparison between two groups. The one-way ANOVA Kruskal–Wallis test was used for comparison of more than 2 groups, followed by Dunn's multiple comparison to compare the mean rank of each column to the mean rank of the control column. Results were expressed as mean ± SD of separate experiments. A *p* value ≤0.05 was considered significant using GraphPad Prism® 8 (GraphPad Software Inc., La Jolla, CA, USA).

## 3. Results

### 3.1. hTERT Expression and Telomerase Activity Are Downregulated in *H. pylori*-Infected Gastric Epithelial Cells

**Figure 2:**
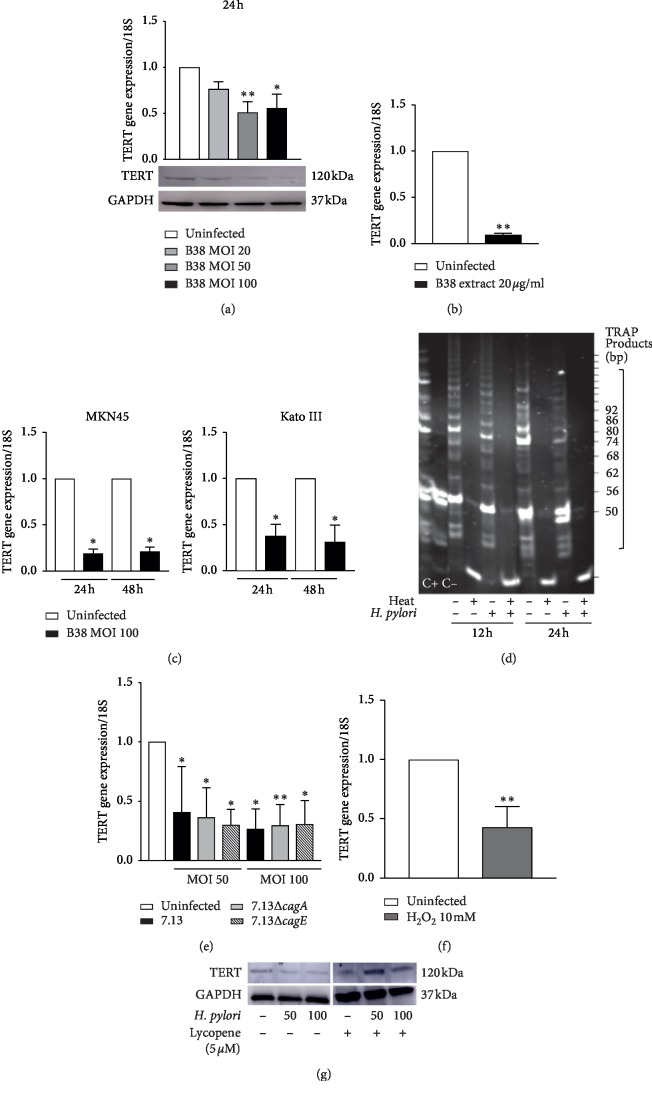
*H. pylori* inhibits *hTERT* gene expression and telomerase activity in gastric epithelial cells. (a) *hTERT* gene expression (upper panel) was measured by real-time qPCR, and protein levels were analysed by western blot (lower panel) in human gastric epithelial cell lines AGS infected with *H. pylori* B38 at MOI 20, 50, and 100 for 24 h. (b) *hTERT* gene expression measured in AGS cells treated with *H. pylori* B38 bacterial extracts (20 *μ*g·ml^−1^) for 24 h. (c) MKN45 and KatoIII gastric epithelial cell lines infected with *H. pylori* B38 for 24 h and 48 h at MOI 100. (d) Telomerase activity analysed by TRAPeze® assay in AGS cell extracts prepared from cells infected with *H. pylori* B38 for 12 h and 24 h (MOI 100). C+, positive control using commercial telomerase-positive cell extracts; C−, negative control. For each analysed condition, heat-inactivated cell extracts obtained after incubation at 85°C for 10 min were also analysed. The displayed gel is representative of 2 independent experiments performed in duplicate. (e) *hTERT* gene expression is also inhibited in AGS cells infected by the *H. pylori* strain 7.13 (MOI 50 and 100) in a CagA- and CagE-independent manner. ^$^*p* < 0.001, one-way ANOVA analysis followed by Dunn's multiple comparison (infected versus uninfected ^*∗*^*p* < 0.05; ^*∗∗*^*p* < 0.01). (f) Oxidative stress generated by exposure of cells to H_2_O_2_ 10 mM for 24 h inhibits the *hTERT* gene expression. (g) Representative western blot of AGS cells infected 24 h with *H. pylori* 7.13 as in (d) and treated with lycopene 5 *μ*M. Lycopene abolished the *H. pylori*-mediated inhibition of TERT. Results are expressed as mean ± SD of three independent experiments (infected versus uninfected ^*∗*^*p* < 0.05; ^*∗∗*^*p* < 0.01; ^*∗∗∗*^*p* < 0.001). *p* < 0.001, one-way ANOVA Kruskal–Wallis followed by Dunn's multiple comparison (infected *versus* uninfected ^*∗*^*p* < 0.05; ^*∗∗*^*p* < 0.01) (a and e).

Human *TERT* (*hTERT*) gene expression was measured by RT-qPCR in the gastric epithelial cell line AGS, infected for 24°h with *H. pylori* strain B38, a clinical isolate from a MALT lymphoma patient [26, 27]. As compared to controls, *hTERT* mRNA levels were decreased in infected cells after 24°h (Figure 2(a), upper panel). A similar inhibition was observed at the TERT protein level (Figure 2(a), lower panel). The same inhibitory effect on *hTERT* gene expression was seen in cells treated with *H. pylori* B38 bacterial extract (20 *μ*g·ml^−1^) for 24°h (Figure 2(b)), suggesting that this downregulation does not require a direct bacterium-epithelial cell interaction and involves one (or more) soluble bacterial factors. The inhibition of *hTERT* gene expression by *H. pylori* was also confirmed in several gastric epithelial cell lines, MKN45 and KatoIII (Figure 2(c)).

Under our experimental conditions of infection, we verified that the decrease of TERT levels was not due to apoptosis. After 24 h and 48°h infection with *H. pylori* strain B38, 77.3% and 70% of the cells were negative for annexin V and 7-aminoactinomycin D staining (live cells), respectively, as compared to 79% and 75.5% in the controls.

We next examined the consequences of *H. pylori* infection on telomerase activity using the Telomeric Repeat Amplification Protocol (TRAP) assay [34], which allows the ability of the telomerase to add telomeric repeats at the 3′end of an oligonucleotide substrate to be determined. As reported in Figure 2(d), a lower telomerase activity was observed when testing the protein extracts of *H. pylori* B38-infected cells, as indicated by the lower intensity and smaller size of the DNA fragments synthesised by these samples, compared to protein extracts from uninfected cells. This effect is particularly observed at 24°h after infection, compared to the pattern of DNA fragments obtained with heat-inactivated samples and samples from the uninfected condition at the same time point (Figure 2(d)). Thus, *H. pylori* infection inhibits *hTERT* gene expression and telomerase activity. As reported in Figure 2(e), *hTERT* gene expression is also downregulated in AGS cells infected with the oncogenic *H. pylori* strain 7.13 [28] and with the isogenic mutants 7.13 ∆*cagA* and 7.13 ∆*cagE* deficient for the oncogenic protein CagA [36] and the virulence factor CagE required for a functional type IV secretion system, respectively [37]. These results indicate that *H. pylori* downregulates *hTERT* gene expression through a CagA- and CagE-independent mechanism.

The production of ROS has been reported during *H. pylori* infection [10, 38, 39] and is confirmed under our experimental conditions in *H. pylori*-infected gastric epithelial cells (Supplementary materials; [Supplementary-material supplementary-material-1]). Similar to *H. pylori* infection, the exposure of cells to hydrogen peroxide (H_2_O_2_) led to a significant decrease of *hTERT* gene expression (Figure 2(f)). In addition, the treatment of cells with lycopene, an efficient singlet oxygen quencher [40], previously shown to prevent ROS production in *H. pylori*-infected cells [31], abolished the inhibitory effect of the infection on the TERT protein levels (Figure 2(g)). These data support that the inhibition of hTERT level during the infection could be regulated by an ROS-mediated mechanism.

### 3.2. Downregulation of *TERT* Gene Expression in *H. Pylori* SS1-Infected Mice Is Associated with Chronic Inflammation

**Figure 3:**
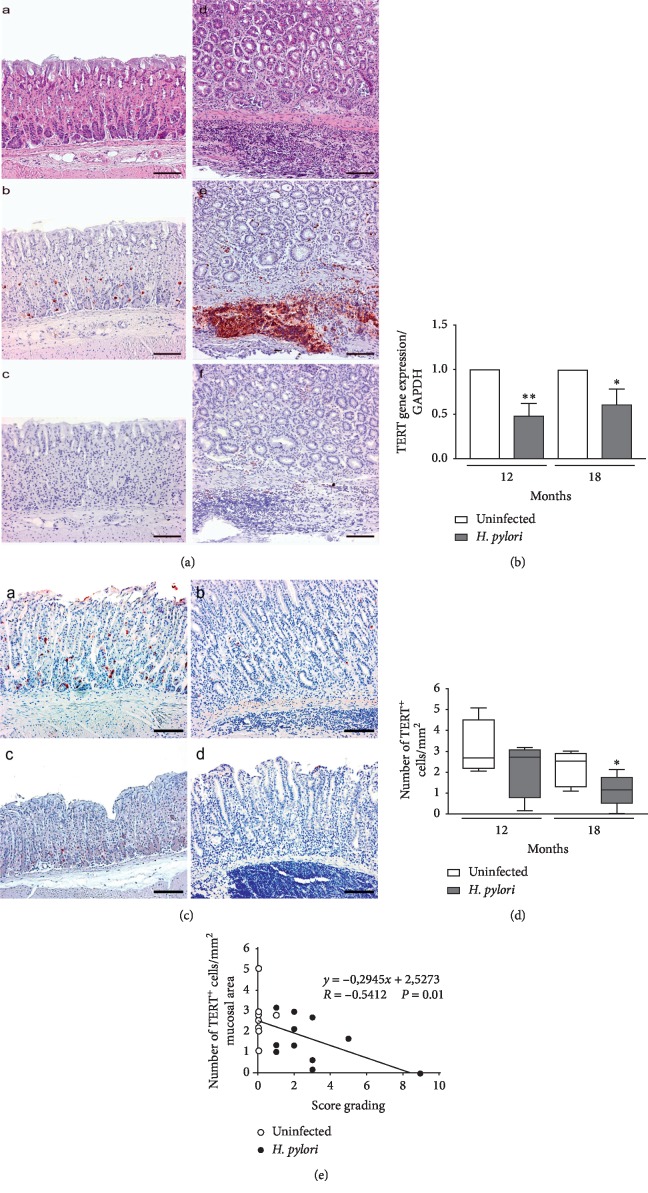
*H. pylori* infection decreases *mTERT* expression in the gastric mucosa of C57BL/6 mice, in the presence of large B lymphocyte aggregates. (a, d) H&E staining and immunostaining of B (b, e) and T (c, f) lymphocytes in gastric sections in infected mice, 12 months after *H. pylori* SS1 infection (d, e, f) and in control mice (a, b, c). Inflammatory infiltrates were observed in the stomach of infected mice, in the lamina propria and submucosa (c). High number of B lymphocytes (e) and a low number of T lymphocytes (f) were present in the inflammatory cell infiltrates in the infected gastric submucosa (e) compared to uninfected (b and c, respectively). Sections of the stomach from the uninfected mice were negative for both B (b) and T (c) lymphocyte staining. Original magnification ×4, bar: 250 *μ*m (a, b, c), and ×10, bar: 100 *μ*m (d, e, f). (b) *mTERT* gene expression in gastric tissues of *H. pylori* SS1-infected mice at 12 and 18 months after infection quantified by real-time qPCR (Taqman). Results are expressed as means ± SD of three independent experiments (infected versus uninfected ^*∗*^*p* < 0.05; ^*∗∗*^*p* < 0.01). (c) TERT immunolabeling in gastric tissue sections from uninfected mice (a, c) and *H. pylori* SS1-infected (b, d) after 12 (a, b) and 18 (c, d) months. Lower TERT staining is observed in the gastric mucosa in the area of the inflammatory B lymphocyte infiltrates in infected samples. Original magnification: ×10, bar: 100 *μ*m (a, b), and ×4, bar: 250 *μ*m (c, d). (d) Number of TERT-positive cells/mm^2^ mucosal area in gastric tissue sections of uninfected and infected samples at 12 and 18 months. Results are expressed as mean ± SD (infected versus uninfected ^*∗*^*p* < 0.05) according to Mann–Whitney analysis. (e) Inverse correlation between the number of TERT-positive cells/mm^2^ mucosal area and the total score grading inflammatory lesions in uninfected (white symbols) and infected mice (black symbol), indicating that TERT level decreases with the exacerbation of gastric inflammation. Each symbol represents one mouse.

We then took advantage of the ability of the *H. pylori* strain SS1 to chronically colonize (i.e., for several months) the gastric mucosa of mice [29], to investigate *mTERT* gene expression in C57BL/6 mice infected for 12 and 18 months. The measure of *H. pylori* gastric colonization loads confirmed that mice were successfully infected (Supplementary materials; [Supplementary-material supplementary-material-1]). Histological analysis of infected stomachs showed active gastritis as indicated by the semiquantitative evaluation of histological score grading of the inflammatory lesions (Supplementary materials; [Supplementary-material supplementary-material-1]), as previously reported under the same conditions of infection [6, 14]. Hyperplastic gastric lesions and more severe metaplasia were observed in mice infected for 18 months (Supplementary materials; [Supplementary-material supplementary-material-1]). Large inflammatory cell aggregates mostly constituted of B lymphocytes, as shown by the antigen B220-positive staining, were observed in the gastric mucosa of mice after 12 and 18 months of infection (Figures 3(a) and 3(e)). In contrast, no T lymphocytes were found in the inflammatory infiltrates (Figures 3(a) and 3(f)).

*mTERT* gene expression was quantified by RT-qPCR in the gastric tissues of mice. *H. pylori* inhibited *mTERT* gene expression after 12 and 18 months of infection (*p* = 0.0028 and *p* = 0.017, respectively) (Figure 3(b)). Under these conditions, immunohistochemistry analysis of TERT on gastric tissue sections (Figure 3(c)) showed a significantly lower number of TERT-positive gastric cells at 18 months (2.8-fold), compared to uninfected mice (Figure 3(d)). It is important to note the absence of TERT staining in the vicinity of the large aggregates of lymphocytes in the gastric mucosa and submucosa, at both 12 and 18 months of infection (Figure 3(c)). In accordance with this, we found that the number of TERT-positive cells is inversely correlated with the inflammatory score grading (Figure 3(e)), suggesting that lower TERT levels correlate with an exacerbation of inflammatory lesions. The *mTERT* gene expression was also investigated in INS-GAS transgenic mice. These mice, which develop gastric neoplasia in the presence of *H. pylori* infection [33], are a powerful tool to study the early events of gastric carcinogenesis associated with the infection. As expected, *H. pylori* SS1-infected INS-GAS mice showed more severe lesions than infected C57BL/6 mice, with atypical gastric hyperplasia and high-grade dysplasia after 8 months (Supplementary materials; Figures [Supplementary-material supplementary-material-1] and [Supplementary-material supplementary-material-1]). At 8 months after infection, *mTERT* gene expression was lower in infected INS-GAS mice as compared to uninfected. These data support that the increase in the severity of gastric lesions is inversely correlated with TERT levels.

### 3.3. Downregulation of *mTERT* Gene Expression during *H. Pylori* Infection Is Mediated by DNA Hypermethylation

A potential link between *H. pylori*-related promoter CpG islands methylation and telomere shortening has been suggested in the gastric mucosa of infected patients [41]. In addition, multiple levels of regulation of *hTERT* gene expression have been previously reported by methylation of CpG islands at its promoter region [42]. In order to determine if DNA hypermethylation could be involved in the *H. pylori*-mediated inhibition of the *TERT* gene expression, we first tested the effect of a pretreatment of AGS cells with 5′-azacytidine, an inhibitor of DNA methylation, before incubation with *H. pylori* B38 extracts. Under this condition, *hTERT* gene expression was restored to control levels in cells exposed to bacterial extracts (Figure 1(a)). These data suggest that *H. pylori-*induced DNA hypermethylation is responsible for the downregulation of *hTERT* gene expression during the infection.

Aberrant DNA methylation is frequently associated with chronic inflammation, as observed in gastritis patients [43]. We investigated, in the mouse model, the DNA methylation status at the promoter region of the *mTERT* gene (Figure 1(b)), on genomic DNA samples extracted from the gastric mucosa of uninfected C57BL/6 mice and mice infected for 18 months with *H. pylori*. Using a promoter PCR methylation assay, two regions of the *mTERT* promoter were analysed including the CpG island (I) in the core-promoter region and an upstream segment containing a canonical E-box (II) (Figure 1(b)). In both cases (I and II), higher amounts of DNA-methylated fragments were observed by PCR amplification in infected mice (3-fold), compared to uninfected mice (Figures 1(c) and 1(d)). These data suggest that, in the presence of gastric chronic inflammation and preneoplastic lesions in mice, *H. pylori* induces DNA hypermethylation at the promoter region of the *mTERT* gene, leading to the downregulation of its expression.

## 4. Discussion

Impaired telomerase activity and shortened telomere length are associated with genetic instability and an increased risk of gastric cancer [44]. Telomerase could constitute an important target during the interaction of *H. pylori* with gastric epithelial cells. In the present study, we demonstrated that *H. pylori* infection leads to inhibition of *TERT* gene expression through DNA hypermethylation and impairment of telomerase activity, in human gastric epithelial cells. The decrease in TERT levels is confirmed in *H. pylori*-infected mice after 12 and 18 months, together with the induction of inflammation and exacerbation of the severity of gastric lesions. These results were also validated in the INS-GAS mouse model that presents *H. pylori*-induced gastric preneoplasia at 8 months after infection. In accordance with our data, previous studies reported that *H. pylori* infection causes telomere shortening [24, 25]. Moreover, *H. pylori* eradication in gastritis patients was shown to result in increased telomere length and telomerase activity [45]. Importantly, a preferential and massive accumulation of DNA damage close to the telomeric regions, associated with the impairment of DNA repair systems, has been reported in *H. pylori-*infected cells and could trigger loss of telomeres [12]. Our data indicate that telomerase dysfunction, resulting in shortened telomere length, can be considered as a key event at the early steps of gastric carcinogenesis during *H. pylori* infection.

Telomerase deficiency and telomere dysfunction have been reported during chronic inflammatory diseases and contribute to inflammation-associated pathogenesis [46, 47] *H. pylori* infection is characterized by an infiltration of polymorphonuclear cells within the gastric mucosa, as observed in infected mice [6]. In previous studies, the pathogenicity of *H. pylori* infection has been shown to be related to chronic inflammation-associated oxidative stress and DNA damage [48]. Both *H. pylori* and inflammatory cells constitute a source of ROS [10]. In the present study, the *TERT* gene expression was found to be decreased in H_2_O_2_-treated gastric epithelial cells *in vitro*, indicating an ROS-mediated downregulation. In accordance with these results, the treatment of *H. pylori*-infected cells with the antioxidant lycopene led to the restoration of TERT mRNA and protein levels. Thus, our findings suggest that the decrease of TERT-positive cells in the gastric mucosa of infected mice might be an ROS-mediated regulation due to the oxidative stress generated during inflammation. In line with this, it was reported that low-grade chronic inflammation in mice can directly promote ROS-mediated telomeric DNA damage, which is repaired less efficiently than elsewhere in the chromosome [49, 50].

DNA methylation plays a key role during the early steps of carcinogenesis [51]. In *H. pylori*-infected individuals, high levels of CpG methylation have been associated with a higher risk of gastric cancer [43, 52, 53]. A potential link between telomere length shortening and promoter CpG island methylation has been described in the gastric mucosa of *H. pylori*-positive patients [41]. Importantly, ROS-induced oxidative stress during chronic inflammation is associated with aberrant DNA hypermethylation of tumor suppressor gene-promoter region [54]. Furthermore, HOCl and HOBr produced by polymorphonuclear cells during inflammation are also able to interact with DNA and promote DNA methylation [55]. Our data show that *H. pylori* induces DNA hypermethylation at the E-box and CpG island present in the core-promoter region of the *mTERT* gene, leading to the downregulation of its expression. However, the shutting down of TERT expression could, in addition, result from indirect mechanisms through DNA methylation of genes coding for transcriptional regulators as tumor suppressors. The transcription factors USF1 and USF2 activate the transcription of *hTERT* through E-box interaction [15, 16]. Indeed, we previously showed that *H. pylori* induces DNA hypermethylation in the promoter region of *USF1* and *USF2* genes, inhibiting their expression, and consequently resulting in diminished USF1/USF2-E-box binding at the *hTERT* promoter [14].

In *H. pylori*-infected mice, we showed a decrease in *mTERT* gene expression as early as the stage of gastritis and the initial development of preneoplastic lesions. Decrease in *TERT* gene expression has direct consequences on telomerase activity, as we observed in *H. pylori*-infected gastric epithelial cells, *in vitro*. Both models of *H. pylori* infection showed the accumulation of DNA damage [6–8], predominantly observed at the ends of chromosomal arms [12]. Importantly, *H. pylori* DNA damage activity is associated with the impairment of DNA repair systems and p53 deficiency [5], and it plays an important role at the origin of genomic translocations and chromosome end fusion observed in gastric tumors [12]. Dysregulation of the DNA repair system and telomerase activity play a pivotal, decisive role in the decision at the cross-road between the preneoplastic stage and cancer development. At premalignant stages, telomerase deficiency is associated with shortened telomeres, leading to chromosomal instabilities, cell cycle arrest, and replicative senescence. This step needs the activation of the p53-mediated DNA damage response. However, during *H. pylori* infection, the p53-mediated DNA damage response is deficient, thus increasing chromosome instabilities and consequently the promotion of tumorigenesis [56]. This mechanism needs the reactivation of telomerase and maintenance of telomere length. Between the premalignant stage and cancer, *TERT* expression is thus reactivated resulting in unlimited cellular proliferation and tumorigenesis [57], as described during hepatocarcinogenesis (HCC) to enable malignant transformation and HCC development [58]. *TERT* expression has been reported to be reactivated in 85% of all cancers [59]. Importantly, reactivation of *TERT* expression is also associated with *TERT*-promoter mutations, currently found in many types of cancers. As an example in melanoma, T > G at −57 base pairs from the transcription start site (TSS) generates an E-twenty-six (ETS) transcription factor-binding site that leads to the upregulation of *TERT* transcription [60]. Therefore, we propose that telomerase deficiency, together with *H. pylori*-induced chronic inflammation, promotes accumulation of chromosome instabilities, driving cell transformation at the earliest stage toward preneoplastic phase. It is during later stages of carcinogenesis that the activation of *TERT* expression and telomerase activity may occur, resulting in an uncontrolled proliferation pattern and tumorigenesis, previously reported in gastric cancer [61, 62].

## 5. Conclusions

In conclusion, our study demonstrates that *H. pylori* infection inhibits *TERT* gene expression through DNA hypermethylation at its promoter region. This downregulation occurs during chronic gastritis and the development of preneoplastic lesions. This regulation is mediated through ROS production induced by the infection and chronic inflammation. The decrease in TERT levels is associated with a progressive shortening of telomeres with direct consequences on cell differentiation and proliferation, thus contributing to the early steps of the gastric carcinogenesis process.

## Data Availability

The data used to support the findings of this study are included within the manuscript.

## Supplementary Materials

Supplementary Materials Figure S1: *H. pylori* induces ROS production in gastric epithelial cells. MKN45 cells treated with H2DCFDA were exposed to increasing concentrations of bacterial extracts (BEs) of *H. pylori* strain 7.13 or to a vehicle control. WT MKN45 (mock) cells were used to assess basal levels of ROS, and H_2_O_2_ (5 mM) was used as a positive control. Intracellular DCF fluorescence (readout of ROS production) was measured using an excitation/emission wavelength of 488/530. Results are expressed as means ± SEM of a representative experiment (*n* = 3). Treated cells compared to vehicle control: ^*∗∗∗*^*p* < 0.001; ^*∗∗∗∗*^*p* < 0.0001. Figure S2: gastric inflammatory and preneoplastic lesions are induced in *H. pylori* chronically infected mice. (a) Quantification of stomach colonization by *H. pylori* SS1 after 12 and 18 months of infection. Each symbol corresponds to a single mouse. (b) Semiquantitative evaluation of histologic lesions induced by *H. pylori* in the gastric mucosa of mice. The microscopic changes (inflammation, hyperplasia, and metaplasia) were semiquantitatively scored on H&E-stained paraffin sections from 0 to 5 according to Eaton and Coll [4]. The scores of inflammation were similar at 12 and 18 months after infection. However, hyperplasia was only observed in mice infected for 18 months; at this stage, histologic lesions are associated with a higher severity of metaplasia as compared to the lesions observed in 12-month infected mice. Infected mice compared to noninfected ^*∗*^*p* < 0.05. Figure S3: *H. pylori* inhibits mTERT gene expression in the gastric mucosa of INS-GAS transgenic mice. INS-GAS transgenic mice were chronically infected with *H. pylori* SS1 for 8 months, and gastric lesions were compared to noninfected mice as described in Materials and Methods. (a) Representative histological changes in gastric mucosa of *H. pylori* infected (b) and noninfected (a) mice. (b) Quantification of *H. pylori* gastric colonization at 8 months after infection. Each symbol corresponds to a single mouse. (c) Semiquantitative evaluation of the histologic lesions induced by *H. pylori* in the gastric mucosa of mice. The microscopic changes (inflammation, hyperplasia, and metaplasia) were scored from 0 to 5 on H&E-stained paraffin sections, according to [65]; Original magnification: ×4, bar: 250 *μ*m (a); ×10, bar: 100 *μ*m (b). The gastric mucosa of noninfected mice was thickened due to the presence of hyperplasia, dilatation of gastric glands, and metaplasia that occur spontaneously in the INS-GAS mice. In the infected mice, the severity of these lesions is higher than in noninfected mice. (d) mTERT gene expression quantified by real-time RT-PCR from RNA isolated from the gastric mucosa of infected and noninfected mice. Values represent the mean ± SEM of three independent measurements for each group of mice. Infected mice compared to noninfected; ^*∗*^*p* < 0.05; ^*∗∗*^*p* < 0.01.
