# Supplementary material for: DNA Hypermethylation Downregulates Telomerase Reverse Transcriptase (TERT) during H. pylori-Induced Chronic Inflammation
Source: J Oncol. 2019 Dec 31;2019:5415761. doi: 10.1155/2019/5415761 (PMC7012206; doi:10.1155/2019/5415761)
Supplement: Supplementary Materials — Figure S1: H. pylori induces ROS production in gastric epithelial cells. MKN45 cells treated with H2DCFDA were exposed to increasing concentrations of bacterial extracts (BEs) of H. pylori strain 7.13 or to a vehicle control. WT MKN45 (mock) cells were used to assess basal levels of ROS, and H2O2 (5 mM) was used as a positive control. Intracellular DCF fluorescence (readout of ROS production) was measured using an excitation/emission wavelength of 488/530. Results are expressed as means ± SEM of a representative experiment (n = 3). Treated cells compared to vehicle control: ∗∗∗p < 0.001; ∗∗∗∗p < 0.0001. Figure S2: gastric inflammatory and preneoplastic lesions are induced in H. pylori chronically infected mice. (a) Quantification of stomach colonization by H. pylori SS1 after 12 and 18 months of infection. Each symbol corresponds to a single mouse. (b) Semiquantitative evaluation of histologic lesions induced by H. pylori in the gastric mucosa of mice. The microscopic changes (inflammation, hyperplasia, and metaplasia) were semiquantitatively scored on H&E-stained paraffin sections from 0 to 5 according to Eaton and Coll [4]. The scores of inflammation were similar at 12 and 18 months after infection. However, hyperplasia was only observed in mice infected for 18 months; at this stage, histologic lesions are associated with a higher severity of metaplasia as compared to the lesions observed in 12-month infected mice. Infected mice compared to noninfected ∗p < 0.05. Figure S3: H. pylori inhibits mTERT gene expression in the gastric mucosa of INS-GAS transgenic mice. INS-GAS transgenic mice were chronically infected with H. pylori SS1 for 8 months, and gastric lesions were compared to noninfected mice as described in Materials and Methods. (a) Representative histological changes in gastric mucosa of H. pylori infected (b) and noninfected (a) mice. (b) Quantification of H. pylori gastric colonization at 8 months after infection. Each symbol corresponds to a single mous [file 5415761.f1.pdf]

Supplementary Material

DNA hypermethylation downregulates Telomerase Reverse Transcriptase (TERT) during *H. pylori*-induced chronic inflammation.

Françoise I. Bussière[1,a], Valérie Michel[1], Julien Fernandes[1,b], Lionel Costa[1,c], Vania Camilo[1,d], Giulia Nigro[3], Hilde De Reuse[1], Laurence Fiette[2,e] and Eliette Touati[1]*

Supplementary Results

Induction of reactive oxygen species (ROS) by *H. pylori*

ROS production was quantified in the human adenocarcinoma gastric epithelial cell line MKN45 incubated for 24h with increasing concentrations (20µg.ml^{-1} to 100µg.ml-1) of total extracts of *H. pylori* 7.13 lysates (bacterial extracts, BE) as indicated in the materials and methods. The production of ROS was assessed using the ROS sensitive fluorescent probe 2',7'-dichlorodihydrofluorescein diacetate (H_2-DCF-DA) as previously described [1]. Under these conditions, *H. pylori* extracts led to a dose-dependent induction of ROS in cells as observed in the presence of H_2O_2 (Figure S1).

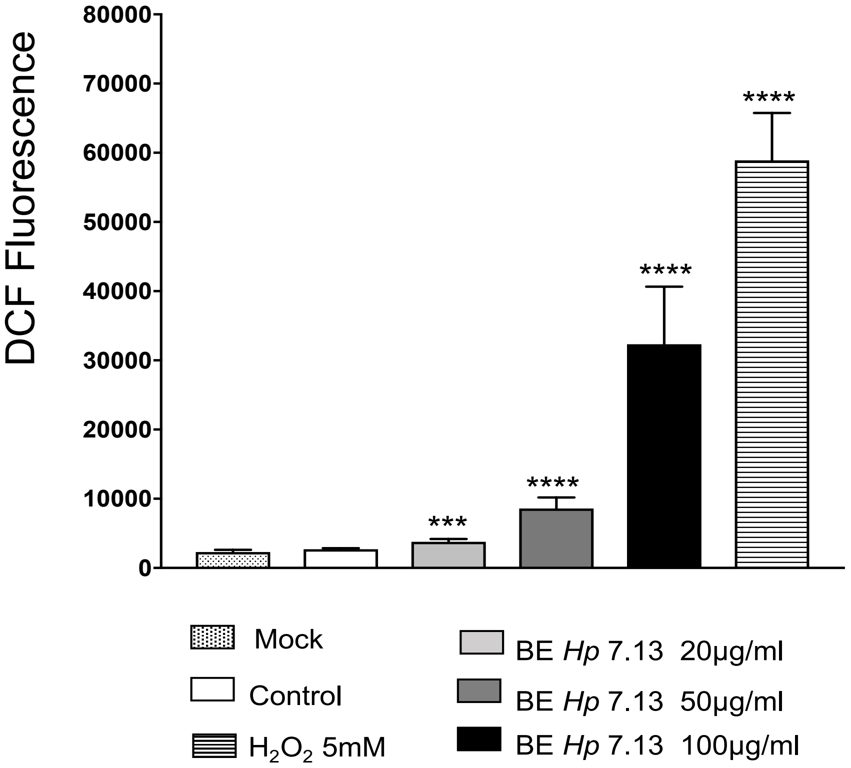

Figure S1: *H. pylori* induces ROS production in gastric epithelial cells. MKN45 cells treated with H_2DCFDA were exposed to increasing concentrations of bacterial extracts (BE) of *H. pylori* strain 7.13 or to a vehicle control. WT MKN45 (mock) cells were used to assess basal levels of ROS and H_2O_2 (5mM) was used as a positive control. Intracellular DCF fluorescence (readout of ROS production) was measured using an excitation/emission wavelength of 488/530. Results are expressed as means ± SD of a representative experiment (n=3).; *P<0.0001*

one-way ANOVA Kruskal Wallis followed by Dunn's multiple comparison: treated cells compared to vehicle control: p<0.05*; p<0.001***

Gastric inflammatory and pre-neoplastic lesions are induced in *H. pylori* chronically infected mice

In order to analyse the *in vivo* relevance of data obtained in gastric epithelial cells *in vitro*, C57BL/6 mice were chronically infected with the *H. pylori* strain SS1, which is able to colonize the mouse stomach for long periods [2]. *H. pylori* gastric colonization was quantified on stomach fragments as previously described [2, 3 expressed as colony forming units (cfu)/g of gastric tissue. As shown in Figure S2a, all mice were still infected after 12 and 18 months. The absence of *H. pylori* gastric colonization in uninfected mice was also confirmed (not shown). At both time points, *H. pylori* induced gastric inflammatory lesions. The histological analysis of infected stomachs showed active gastritis as already reported in Figure 2a. The histological score grading of the inflammatory lesions were evaluated according to Eaton and coll [4]. Briefly, inflammation corresponding to infiltrates of polymorphonuclear cells (PMN) and plasmocytes was graded as follows: 0-no infiltrates; 1 mild-multifocal infiltration; 2 mild-widespread infiltration; 3- Mild, widespread and moderate multifocal infiltration; 4- moderate widespread infiltration; 5-moderate, widespread and severe multifocal infiltrations. The scores of inflammation were similar at 12 and 18 months post-infection (Figure S2b). However, hyperplasia was only observed in mice infected for 18 months; as previously reported [5, 6], at this stage a higher severity of metaplasia was observed as compared to 12 months infected mice.

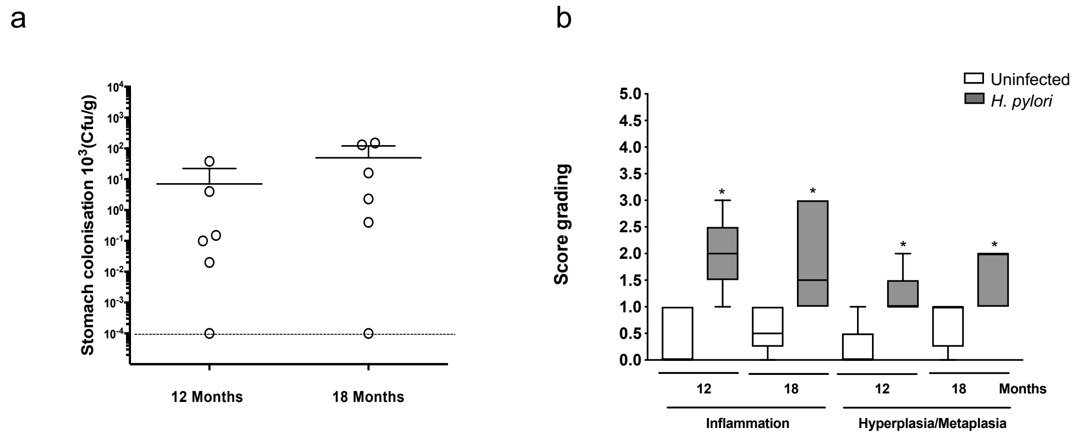

Figure S2

Figure S2: Gastric inflammatory and pre-neoplastic lesions are induced in *H. pylori* chronically infected mice. a) Quantification of stomach colonization by *H. pylori* SS1 after 12 and 18 months of infection. Each symbol corresponds to a single mouse. **b)** Semi-quantitative evaluation of histologic lesions induced by *H. pylori* in the gastric mucosa of mice. The microscopic changes (inflammation, hyperplasia and metaplasia) were semi-quantitatively scored on H&E stained paraffin sections from 0 to 5 according to Eaton and coll [4]. The scores of inflammation were similar at 12 and 18 months post-infection. However, hyperplasia was only observed in mice infected for 18 months; at this stage, histologic lesions are associated with a higher severity of metaplasia as compared to the lesions observed in 12 months infected mice. Infected mice compared to uninfected $p<0.05$*.

H. pylori inhibits *mTERT* gene expression concomitantly to the development of gastric preneoplasia in the INS-GAS mouse model

INS-GAS mice are transgenic for the human gastrin. The presence of *H. pylori* infection has been reported to exacerbate the development of gastric intraepithelial neoplasia in these mice which constitute a suitable model for the study of the early events at the origin of gastric cancer [7, 8]. As described in materials and methods, INS-GAS mice were orogastrically infected with the *H. pylori* strain SS1 for 8 months. The presence of the infection was confirmed in all mice (Figure S3b). At this time-point, uninfected mice showed gastritis, epithelial defects with gland

dilatation and glandular hyperplasia (Figure S3a(a)), as also indicated by the score grading of the lesions (Figure S3c). More severe gastric inflammation was observed in infected mice with the presence of significant aggregates of inflammatory cells in the submucosa, hyperplasia and intestinal metaplasia, associated with a greater thickness of the gastric mucosa compared to uninfected mice (Figure S3a). Under this condition, the *mTERT* gene expression is inhibited in *H. pylori*-infected mice after 8 months, as compared to uninfected mice (Figure S3d). These data indicate that the *H. pylori*-mediated inhibition of TERT levels occurs concomitantly with the development of gastric preneoplasia.

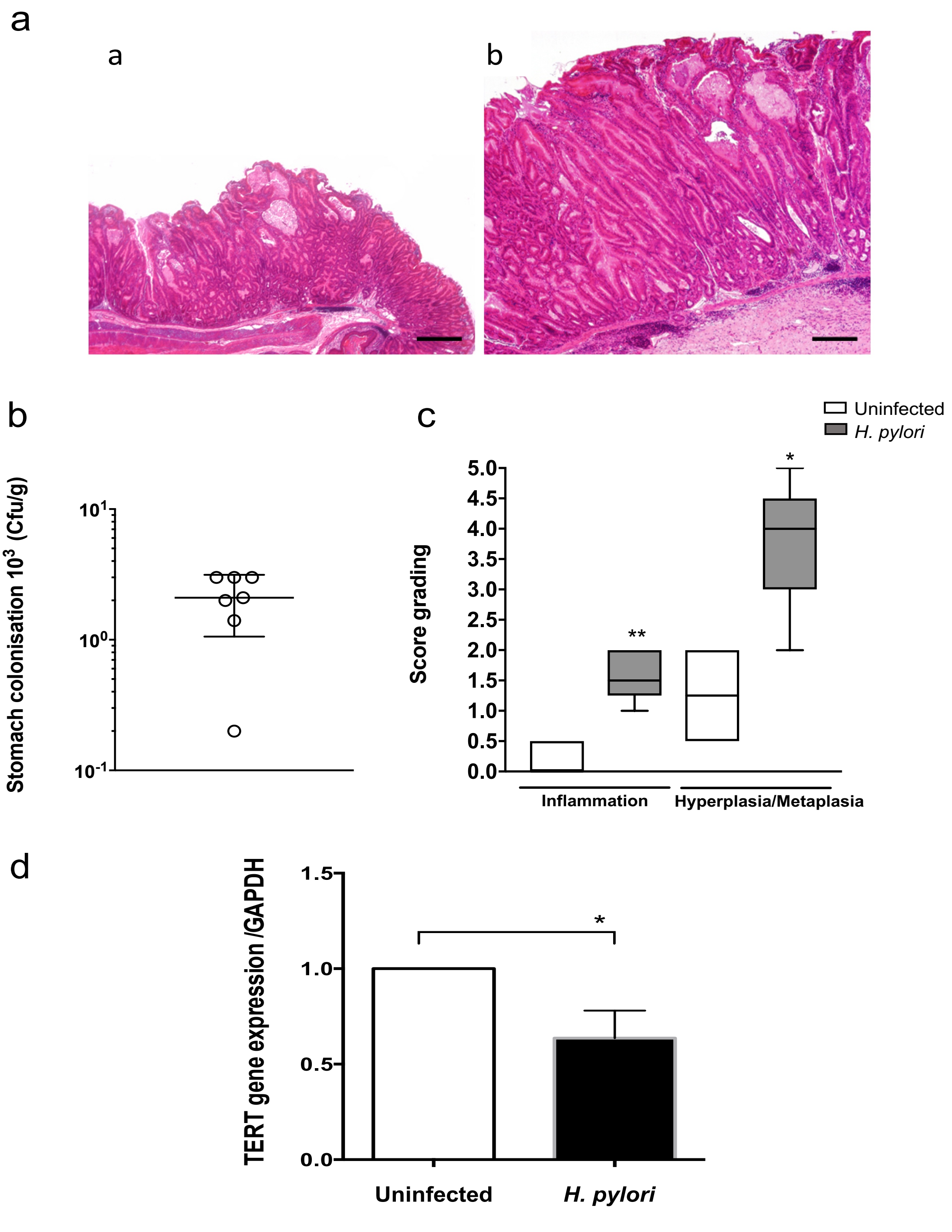

Figure S3: *H. pylori* inhibits *mTERT* gene expression in the gastric mucosa of INS-GAS transgenic mice. INS-GAS transgenic mice were chronically infected with *H. pylori* SS1 for 8 months and gastric lesions were compared to uninfected mice as described in materials and methods. **a)** Representative histological changes in gastric mucosa of *H. pylori* infected (b) and uninfected (a) mice. **b)** Quantification of *H. pylori* gastric colonization at 8 months post-infection. Each symbol corresponds to a single mouse**. c)** Semi-quantitative evaluation of the histologic lesions induced by *H. pylori* in the gastric mucosa of mice. The microscopic changes (inflammation, hyperplasia and metaplasia) were scored from 0 to 5 on H&E stained paraffin

sections. Original magnification: ×4, bar: 250µm (a); ×10, bar: 100µm (b). The gastric mucosa of uninfected mice was thickened due to the presence of hyperplasia, dilatation of gastric glands and metaplasia that occur spontaneously in the INS-GAS mice. In the infected mice, the severity of these lesions is higher than in uninfected mice. **d)** *mTERT* gene expression quantified by real-time RT-qPCR from RNA isolated from the gastric mucosa of infected and uninfected mice. Values represent the mean±SD of three independent measurements for each group of mice. Infected mice compared to uninfected $p<0.05$*; $p<0.01$** using the Mann-Whitney test.

References

1. Ding SZ, Minohara Y, Fan XJ, Wang J, Reyes VE, Patel J, Dirden-Kramer B, Boldogh I, Ernst PB, Crowe SE. 2007. *Helicobacter pylori* infection induces oxidative stress and programmed cell death in human gastric epithelial cells. *Infect Immun* 75:4030-4039.
2. Lee A, O'Rourke J, Ungria M, Robertson D, Daskalopoulos G, Dixon M. 1997. A standardized mouse model of *Helicobacter pylori* infection : introducing the Sydney strain. *Gastroenterology* 112:1386-1397.
3. Ferrero R, Thiberge J, Huerre M, Labigne A. 1998. Immune responses ofspecific-pathogen-free mice to chronic *Helicobacter pylori* (strain SS1) infection. *Infect Immun* 66:1349–1355.
4. Eaton A, Danon J, Krakowka S, Weisbrode E. 2007. A reproducible scoring system for quantification of histologic lesions of inflammatory disease in mouse gastric epithelium. Comp Med 57(1): 57–65.
5. Touati E, Michel V, Thiberge J, Wuscher N, Huerre M, Labigne A. 2003. Chronic *Helicobacter pylori* infections induce gastric mutations in mice. *Gastroenterology* 124:1408-1419.
6. Bussière FI, Michel V, Memet S, Ave P, Vivas JR, Huerre M, Touati E. 2010. *H. pylori*-induced promoter hypermethylation downregulates USF1 and USF2 transcription factor gene expression. *Cell Microbiol* 12:1124-1133.
7. Wang TC, Dangler C, Chen D, Goldenring J, Koh T, Raychowdhury R, Coffey R, Ito S, Varro A, Dockray G, Fox J. 2000. Synergistic interaction between hypergastrinemia and Helicobacter infection in a mouse model of gastric cancer. *Gastroenterology* 118:36-47.
8. Fox JG, Rogers AB, Ihrig M, Taylor NS, Whary MT, Dockray G, Varro A, Wang TC. 2003. *Helicobacter pylori* associated gastric cancer in INS-GAS mice is gender specific. *Cancer Res* 63: 942-950.
